# Extracts from the Minutes of Atlanta Academy of Medicine

**Published:** 1871

**Authors:** 


### *Extracts from the Minutes of Atlanta Academy of Medicine.*

May 19, 1871.

Dr. W. F. Westmoreland.—An adult placed under my care, has suffered for three days with constant hæmoptysis. The patient has had previous attacks, and probably has tuberculous deposit in the lungs. I desire the opinion of members as to the most successful manner of treating the hemorrhage, and for this purpose have reported the case. Do not hemorrhagic consumptives live longer than those who lose no blood?

Dr. H. V. M. Miller. — This case suggests an important inquiry. Why is it that one in vigorous health should be thus affected? Twenty-five years ago, I would have had no doubt of arresting the hemorrhage by blood-letting, acetate lead, opium, etc. Now, I would hesitate to bleed, from the fear of hastening the ulterior result to be expected in the present state of advancement in medical knowledge. Acetate of lead certainly acts favorably by increasing the coagulability of the blood, and is doubtless a useful hœmostatic in this and other forms of internal hemorrhage. There is even now, perhaps, no good reason for failing to abstract blood from plethoric subjects with hæmoptysis. Less prostration and debility will probably result from the sudden loss of a pint of blood in the arrest of the existing hemorrhage, than would result from the constant drain if suffered to continue.

Dr. Harden.—Veratrum, in all probability, would be serviceable in the treatment of this hemorrhage, by lessening the force of the circulation, as a sedative to the heart. As a direct styptic carbolic acid is, perhaps, preferable to any other remedy in hemorrhage. From my observation of its effect in hematuria, it is exactly suited to cases of internal hemorrhage of any kind. In the dose of two grains, dissolved in two or three ounces of water, and given every four hours, the discharge of bloody urine, was promptly arrested in a case under my care.

Dr. Love.—I desire to call Dr. Westmoreland's attention to the action of ergot in hemorrhage. It has been found useful in passive hemorrhage, and there are good reasons for believing that it may be useful in the case under consideration.

In a case of hæmoptysis, a report of which recently came under my observation, in a Medical Journal, was greatly benefitted by inhaling powdered persulphate of iron, and if what is claimed for this treatment be true this remedy might prove effectual when used for this purpose by inhalation.

Dr. Miller.—I desire an explanation of the styptic action of ergot in hemorrhage from the lungs. The manner in which uterine hemorrhage is arrested by ergot, is very well understood. The remedy produces contraction of the uterus, and thus mechanically arrests the further flow of blood. No such action being had in connection with the lungs, if the flow of blood is suppressed at all, it must be done by some other mode of action. The persulphate of iron is an efficient styptic when applied to the part from which the blood exudes, and will doubtless arrest hæmoptysis if it can be applied to the bleeding tissue.

Dr. Love.—An explanation of the action of ergot as a styptic is found, in its effects upon the capillaries, in the production of dry gangrene. This death of the part results from arrest of circulation through the small blood-vessels, and it is probable that in this way hemorrhage may be arrested in the lungs, since reports have been made of such constringing action, by the remedy in spinal meningeal irritation and kidney affections.

Dr. Miller.—The theory which ascribes dry gangrene to closure of the capillaries, I do not endorse. No such condition is found to exist, and therefore, when the death of a part results from the use of ergot, its *modus operandi* must be sought in some other direction. We have no good reason for supposing that ergot will arrest hæmoptysis. I have never seen any such effect from it, and as conclusions are sometimes hastily arrived at, errors may be promulgated by unreliable statistical reports. To be worthy of confidence, reported effects of remedies must be sustained by numerous successive instances of the same result.

Dr. Westmoreland.—Senile gangrene is probably the result of capillary obstruction, caused by fibrin separated from the blood by the rough ossified interior of the arteries upon which it accumulates, and when washed off by the mass of blood, fills up the capillaries so as to prevent nourishment of the tissues.

---

May 26.

Dr. J. M. Boring.—On May 2d was called to see a white boy eight years old, healthy and well grown, and whose right heel had been punctured by a pin, to a considerable depth. The patient was first seen at 5 p. m. The face was flushed ; the left leg swollen and of purplish, erysipelatous hue ; fever high, with slight delirium.

The history given by the parents is, that four days previously, the wound had been made. The pin having been withdrawn at once, no inconvenience was experienced for two days, when the punctured part became painful, and symptoms of fever, with restlessness at night, were discovered. The next morning, May 1, and third day after the injury, he seemed better—got up as usual, but complained of pain and soreness of the ankle, knee and groin of the opposite side from that receiving the injury. He was unable to sit up long. Fever and slight delirium came on, with increase of pain throughout the whole extent of the left leg. The wound in the heel of the right foot had been opened, the night previously, and discharged bloody water, as described by his mother.

The next day, May 2nd, the symptoms increased in violence, and at the time of our first visit, presented the symptoms before mentioned.

I advised the following, and left, promising to return next morning :

℞.
Hydrag. Chlorid. Mite. grs. viij.
Pulvis Doveri grs. vi.
M. et. ft. Pulv. No. iv.
One to be given every four hours.

Also, as a local application :

℞
Tinct. Arnica f℥ij.
Tinct. Opii f℥j.
M.
To be applied freely to the whole limb.

At 1 o'clock, A. M., was called again, in haste, and found him speechless; bathed in perspiration, in great agony, and with slight spasms of the superior extremities.

I immediately requested the counsel of Dr. W. F. Westmoreland, who arrived about 2 o'clock. On consultation, the treatment directed on previous afternoon was continued, with the under standing that we should meet again at 7 A. M.

7 o'clock, A. M.—Found the patient evidently sinking, notwithstanding the free use of brandy; he continued to decline, and died at half-past 10 o'clock, A. M.

This case presents some striking peculiarities. The trifling injury in the heel of the right foot, seemed to have given rise to the painful and rapidly fatal disease, the precise nature of which I am unable, satisfactorily, to determine. Did the patient die rom tetanus, erysipelas or pyæmia?

Dr. Moore.—A case was recently presented for treatment, with febrile excitement, from a puncture in the foot by a nail. No other unfavorable symptom occurred, and the patient recovered under the use of gelseminum and a mercurial.

Dr. J. Stainback Wilson.—There is nothing in the history of Dr. Boring's case, that shows decidedly tetanic symptoms. Pyæmia would most likely give rise to the alarming condition described in this case, and to this poisoned condition of the blood should probably be attributed the rapidly fatal tendency observed.

Dr. Harden.—In a case of spider-bite once under my care, there was exhibited some of the peculiarities noticed in the case reported by Dr. Boring. The symptoms of excruciating pain, etc., attending this form of poisoning, were manifested in the limb opposite that wounded by the insect.

Dr. W. F. Westmoreland.—In the case reported by Dr. Boring, which I visited with him, the marked prominence of the the veins, and lymphatics, and other evidences of disease in these vessels, leads me to the conclusion that probably the fatal termination depended upon this diseased condition. The usual symptoms of tetanus were wanting in the case, and there seems to be no good reason for supposing that such impression had been made upon the cerebro-spinal nervous centre, as develops tetanic spasm.

Dr. Miller-—The true pathological condition, in Dr. Boring's case, is somewhat difficult to determine. Some more definite opinion might have been arrived at by seeing the patient. Could it have been tetanus? The symptoms as given, do not seem to justify this opinion. Was the case one of traumatic erysipelas? Usually, the symptoms of this disease are different from those detailed in the case reported. After hearing all that has been said on the subject, the inclination of my mind is that to pyæmia is attributable the fatal termination.

The objections that may be made to this opinion are, that the amount of pus was too small, and the usual conditions favoring absorption of this substance were not present. No bones were broken, nor was there such condition of the veins favoring its absorption, as is found in a recently delivered uterus. On the other hand, it is known that pyæmia may prove fatal without a large amount of pus being taken into the blood. It seems to act as leven, poisoning the whole mass of circulating fluid, and thus deisturb nutrition. Inflammation in the opposite limb from that injured, can be accounted for only on the supposition that the blood was poisoned.

There is a similarity between milk-leg and the case under consideration. The former occurs under circumstances favoring the absorption of pus, owing to the exposed condition of the vessels before the uterus has firmly contracted. after delivery.

In the symptoms detailed in Dr. Boring's case, there seems to be a striking similarity to those I have witnessed in the painful condition of the leg alluded to, occurring with lying—in women.

A case of pyæmia once came under my observation, in which the peculiarity observed in Dr. Boring's case, was also found. The bone of one leg was injured, giving rise to the absorption of pus from the exposed condition of the vessels, and the poisonous effects were manifested in the opposite limb.

Dr. W. F. Westmoreland.—The pathology of milk-leg, given by Dr. Miller, is not, to my mind, very well sustained by the usual symptoms found in this disease. While in the case reported to-night, there may be a very striking resemblance to milk-leg, yet in both, the usual symptoms of pyæmia are wanting. Rigors are supposed to afford the most certain evidence of this poisoned condition of the blood. In milk-leg and in the case reported, this does not seem to be a leading symptom. While I am not very decided in opinion as to the cause of derangement in the case seen with Dr. Boring, yet the most reasonable view seems to be that a kind of erysipelatous inflammation of the vessels of the limb existed from the absorption of unhealthy fluid, probably not pus. As to the pathology of milk-leg, I have found nothing more reasonable than that advanced by the lamented Dr. Coe, of Atlanta, several years ago. He advocated the opinion that the symptoms were the result of reflex-nervous impression.

Dr. Wilson.—The views entertained by Dr. Miller seem reasonable, but while injury to bones may favor the entrance of pus into the veins, yet the absorption certainly occurs often under other circumstances.

That rigors are generally felt by pyæmic patients, and make an important diagnostic symptom, as insisted on by Dr. Westmoreland, is also a well established fact.

